# Spermatogonia Loss Correlates with LAMA 1 Expression in Human Prepubertal Testes Stored for Fertility Preservation

**DOI:** 10.3390/cells10020241

**Published:** 2021-01-27

**Authors:** Magdalena Kurek, Elisabet Åkesson, Masahito Yoshihara, Elizabeth Oliver, Yanhua Cui, Martin Becker, João Pedro Alves-Lopes, Ragnar Bjarnason, Patrik Romerius, Mikael Sundin, Ulrika Norén Nyström, Cecilia Langenskiöld, Hartmut Vogt, Lars Henningsohn, Cecilia Petersen, Olle Söder, Jingtao Guo, Rod T. Mitchell, Kirsi Jahnukainen, Jan-Bernd Stukenborg

**Affiliations:** 1 NORDFERTIL Research Lab Stockholm, Childhood Cancer Research Unit, Department of Women’s and Children’s Health, Karolinska Institutet, and Karolinska University Hospital, 171 64 Solna, Sweden; Elizabeth.oliver@ki.se (E.O.); yanhua.cui@ki.se (Y.C.); jpa51@cam.ac.uk (J.P.A.-L.); cecilia.petersen@ki.se (C.P.); olle.soder@ki.se (O.S.); kirsi.jahnukainen@ki.se (K.J.); 2 Division of Neurogeriatrics, Department of Neurobiology Care Sciences & Society, Karolinska Institutet, 141 83 Huddinge, Sweden; elisabet.akesson@ki.se; 3 The R & D Unit, Stockholms Sjukhem, 112 19 Stockholm, Sweden; 4 Department of Biosciences and Nutrition, Karolinska Institutet, 141 83 Huddinge, Sweden; masahito.yoshihara@ki.se; 5 Center of Neurodevelopmental Disorders (KIND), Department of Women’s and Children’s Health, Karolinska Institutet, Centre for Psychiatry Research, Region Stockholm and Astrid Lindgren Children’s Hospital, Karolinska University Hospital, 171 64 Solna, Sweden; martin.becker@ki.se; 6 Children’s Medical Center, Landspítali University Hospital, 101 Reykjavik, Iceland; ragnarb@landspitali.is; 7 Department of Paediatrics Faculty of Medicine, University of Iceland, 101 Reykjavik, Iceland; 8 Department of Paediatric Oncology and Haematology, Clinical Sciences, Lund University, Barn-och Ungdomssjukhuset Lund, Skånes Universitetssjukhus, 221 85 Lund, Sweden; Patrik.Romerius@skane.se; 9 Division of Paediatrics, Department of Clinical Science, Intervention and Technology, Karolinska Institutet, 141 52 Huddinge, Sweden; mikael.c.sundin@sll.se; 10 Pediatric Blood Disorders, Immunodeficiency and Stem Cell Transplantation Unit, Astrid Lindgren Children’s Hospital, Karolinska University Hospital, 141 86 Huddinge, Sweden; 11 Division of Paediatrics, Department of Clinical Science, Umeå University, 901 87 Umeå, Sweden; Ulrika.Noren.Nystrom@regionvasterbotten.se; 12 Department of Paediatric Oncology, The Queen Silvia Children’s Hospital, 416 50 Gothenburg, Sweden; cecilia.langenskiold@vgregion.se; 13 Crown Princess Victoria’s Child and Youth Hospital, and Department of Biomedical and Clinical Sciences, Linköping University, 581 83 Linköping, Sweden; hartmut.vogt@liu.se; 14 Division of Urology, Institution for Clinical Science Intervention and Technology, Karolinska Institutet, 141 52 Huddinge, Sweden; lars.henningsohn@ki.se; 15 Division of Urology, Department of Surgery, University of Utah School of Medicine, Salt Lake City, UT 84112, USA; Jingtao.Guo@hci.utah.edu; 16 MRC Centre for Reproductive Health, The Queen’s Medical Research Institute, The University of Edinburgh, Edinburgh EH16 4TJ, UK; Rod.Mitchell@ed.ac.uk; 17 Edinburgh Royal Hospital for Sick Children, Edinburgh EH9 1LF, UK; 18 Division of Haematology-Oncology and Stem Cell Transplantation, Children’s Hospital, University of Helsinki, Helsinki University Central Hospital, 00029 Helsinki, Finland

**Keywords:** germ cells, basal membrane, stem cell niche, seminiferous tubules, infertility, late effects, spermatogonia, Sertoli cells

## Abstract

Fertility preservation for male childhood cancer survivors not yet capable of producing mature spermatozoa, relies on experimental approaches such as testicular explant culture. Although the first steps in somatic maturation can be observed in human testicular explant cultures, germ cell depletion is a common obstacle. Hence, understanding the spermatogonial stem cell (SSC) niche environment and in particular, specific components such as the seminiferous basement membrane (BM) will allow progression of testicular explant cultures. Here, we revealed that the seminiferous BM is established from 6 weeks post conception with the expression of laminin alpha 1 (LAMA 1) and type IV collagen, which persist as key components throughout development. With prepubertal testicular explant culture we found that seminiferous LAMA 1 expression is disrupted and depleted with culture time correlating with germ cell loss. These findings highlight the importance of LAMA 1 for the human SSC niche and its sensitivity to culture conditions.

## 1. Introduction

Cancer treatment strategies such as chemotherapy and radiotherapy often result in the depletion of the spermatogonial stem cell (SSC) pool leading to lifelong infertility. With increasing numbers of long-term cancer survivors, the preservation of gonadal function and fertility has now become an important focus. While sperm cryopreservation prior to therapy is a common practice for adult males, immature testicular tissue cryopreservation is the only option available for those unable to produce spermatozoa [1]. As a result, current experimental approaches are largely focused on establishing in vitro spermatogenesis from immature testicular tissue [2,3,4]. In 2011, Sato et al. reported the in vitro maturation of fertilisation-competent spermatozoa using culture of intact immature neonatal mouse testicular tissue. While this has yet to be replicated in primates, human testicular explant models have shown promising results, demonstrating steroidogenic functionality of Leydig cells and maturation of Sertoli cells. Despite this, further progression is limited by the significant loss of germ cells, including SSCs, observed during culture [2,3,4]. However, given the success in rodents [5,6], it is likely that the disruption of the somatic environment in humans, which differs from that described in rodents, underlies the observed loss of germ cells.

Spermatogenesis ultimately revolves around the self-renewal and differentiation of SSCs, which are supported by specialised somatic cell populations and a seminiferous basement membrane (BM) that form the SSC niche [7]. Formation of the SSC niche and establishment of the germ cell population begins during early embryonic life. The interaction between the somatic support cells and germ cells (at this stage called gonocytes) becomes established, followed by the organisation of seminiferous cords. Postnatally, gonocytes migrate to the BM becoming spermatogonia. Maturation of the somatic environment continues throughout development to support the changing requirements of the germ cells from prenatal life through to adulthood. Although the exact components of the SSC niche have yet to be defined, the Sertoli cells and the seminiferous BM, which are in direct contact with the SSCs, are generally recognised as key elements [8]. Enclosing the seminiferous tubules, the BM is in direct contact with both the spermatogonia and Sertoli cells. Cellular signalling resulting from cell-basement membrane interactions with Sertoli cells has been shown to mediate positive effects of extra cellular matrix (ECM) components on Sertoli cell morphology, function, differentiation, and cell growth, in addition to directly interacting with SSCs [9]. Additionally, peritubular cells, surrounding the interstitial area of the seminiferous BM, as well as Leydig cells and the vasculature in the interstitium, can be considered a part of the SSC niche, contributing various growth factors and signalling molecules during testicular development and spermatogenesis [10,11].

Although considered an integral part of the SSC niche, little is known about the human testicular BM composition. Composed of extracellular matrix proteins such as fibronectin, type I, IV, VI, and XXI collagens, as well as two of the five laminin α (LAMA) chains, LAMA 1 and 5, the BM provides both structural support and mediates local cell signalling effects through binding and release of growth factors to regulate the niche [12].

LAMA 5 is the most abundant LAMA chain in the adult, abundantly expressed during development and playing a crucial role in stem cell maintenance [13,14,15]. LAMA 1, on the other hand, has shown to be important for the three-germ layer formation in embryonic development, as well as tubulogenesis of kidney and lung [14,15]. LAMA 1 has also been shown to be expressed in the Reichert’s membrane of rodents located adjacent to the derivation point of primordial germ cells [16]. Despite the importance of laminins in development, a detailed description of the laminin composition of the developing human male gonad has yet to be reported. This would contribute to the understanding of the composition and maturation of the SSC niche during human development and therefore, give us greater insight into the key mechanisms of germ cell maintenance.

Here, we report LAMA 1 and type IV collagen to be the key components of the seminiferous BM throughout development, from prenatal life through to adulthood. Further, we highlight the importance of seminiferous LAMA 1 expression in the SSC niche by revealing germ cell depletion upon the loss of LAMA 1 expression in human prepubertal testicular explant cultures.

## 2. Results

### 2.1. LAMA 1 and Type IV Collagen are the Main Components of the Pre- and Postnatal Seminiferous BM

Existing studies on testicular BM proteins have focused on rodent models [17,18,19,20] or human tissue obtained from adult post mortem [21], tumour [22], azoospermic [23], Sertoli-cell-only [21,23,24], or cryptorchid [25] patients. Many of the older studies have utilised primary antibodies that have, in the course of time, been proven to recognize different ECM isoforms from those that were initially intended [22,26]. Given the previous observation of type I, IV, VI, and XXI collagens, fibronectin and LAMA 1 and 5, laminin β (LAMB) 2 and γ (LAMC) 1 in the adult testicular ECM by mass spectrometry [12], we examined the immunolocalization of type I, IV, and VI collagens, fibronectin, and all five LAMA chains during testis development using both human pre- and postnatal tissue samples.

**Figure 1:**
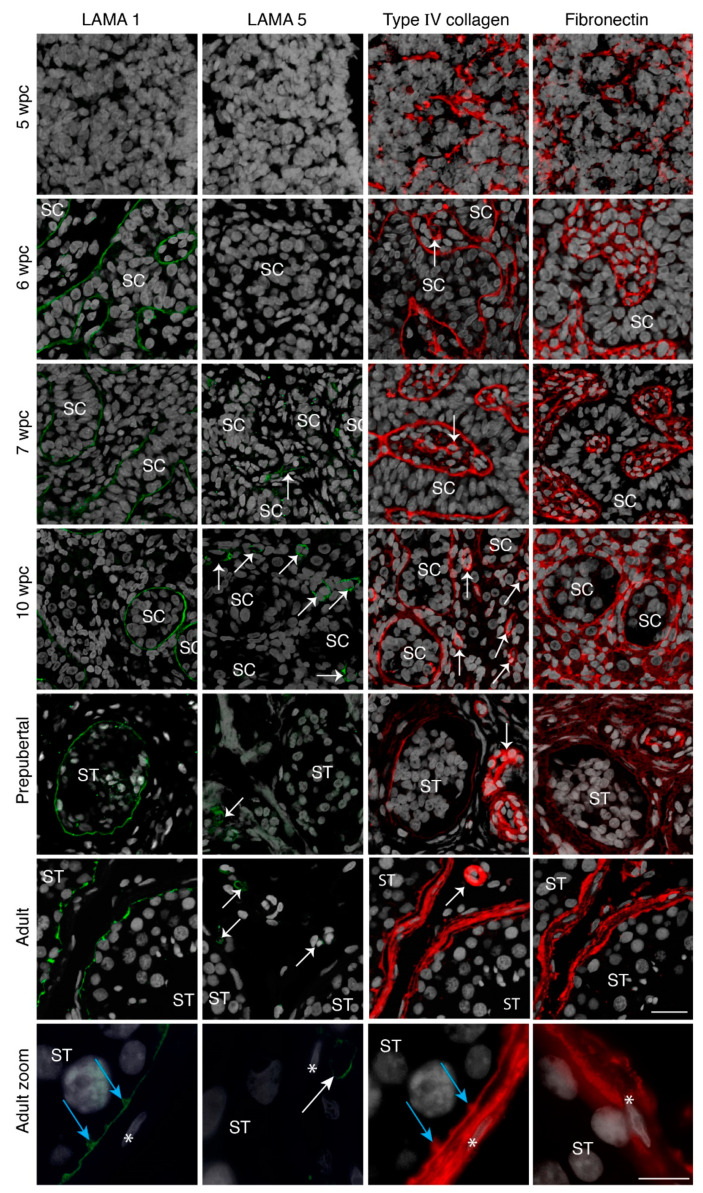
Basement membrane expression of prenatal gonadal and postnatal testicular tissue. Laminin alpha 1 (LAMA 1) (green staining) expression is shown from 6 wpc onwards in the seminiferous basement membrane (BM), while LAMA 5 (green staining) can be observed only in the vasculature from 7 wpc onwards. Type IV collagen (red staining) and fibronectin (red staining) expression is noted from 5 wpc as a net-like structure in the seminiferous BM and also the interstitium in prenatal samples from 6 wpc onwards. Prenatal sample *n* = 27. Prepubertal testicular samples show LAMA 1 expression in the seminiferous BM while LAMA 5 can be observed in the vasculature. Type IV collagen and fibronectin is noted in the seminiferous BM, the vasculature and the interstitium. Prepubertal sample *n* = 16. Adult testicular samples show specialization with LAMA 1 expression in the innermost BM, type IV collagen expression throughout the entire BM, and fibronectin expression in the peritubular BM layer. Adult sample *n* = 3. Counterstain with DAPI (grey staining). SC depicts the seminiferous cord, ST depicts seminiferous tubules, white arrows indicate blood vessels, blue arrows show innermost BM protrusions between tubular cells, * indicate peritubular cells, scale bar: 25 μm, zoom scale bar: 10 µm.

Type I, IV, and VI collagens, as well as fibronectin were observed as the first ECM components as early as 5 weeks post conception (wpc) (Figure 1 and Figure S1). A net-like expression pattern was observed throughout the whole gonad prior to the formation of structured seminiferous cords (Figure 1 and Figure S1). Seminiferous cords were clearly observed from 6 wpc, as revealed by the expression of type I, IV, and VI collagens and fibronectin in the establishing seminiferous BM, as well as throughout the interstitial compartment which persisted until 17 wpc (Figure 1 and Figure S1).

Although laminins consist of each one of the three α (1–5), β (1–3), and γ (1–3) chains, we focused our immunolocalization approach on the detection of α chains whose C-terminal regions are the main facilitators of cell-membrane adhesion and signalling [27,28].

In parallel to the establishment of the basement membrane, LAMA 1 expression could be observed in the seminiferous BM from 6 up to 17 wpc (Figure 1 and Table S1). Interestingly, we were unable to detect any expression of LAMA 2, 3, 4, and 5 in the BM of the seminiferous cords or the interstitial compartment of any prenatal samples, with the exception of vascular LAMA 5 observed from 7 wpc and occasional vascular LAMA 4 expression (Figure 1 and Figure S1).

In order to investigate the potential cell source of the observed protein expression, we examined the gene expression dynamics of previously published single-cell transcriptomics data from human prenatal (4–25 wpc) and postnatal (1–25 years) gonadal germ and somatic cells, as detailed in the Methods Section. Expression patterns of type I (*COL1A1*), IV (*COL4A1*), and VI (*COL6A1*) collagens, fibronectin (*FN1*), and all three laminin chains *LAMA 1–5*, *LAMB 1–3*, and *LAMC 1–3* [29,30] were evaluated.

**Figure 2:**
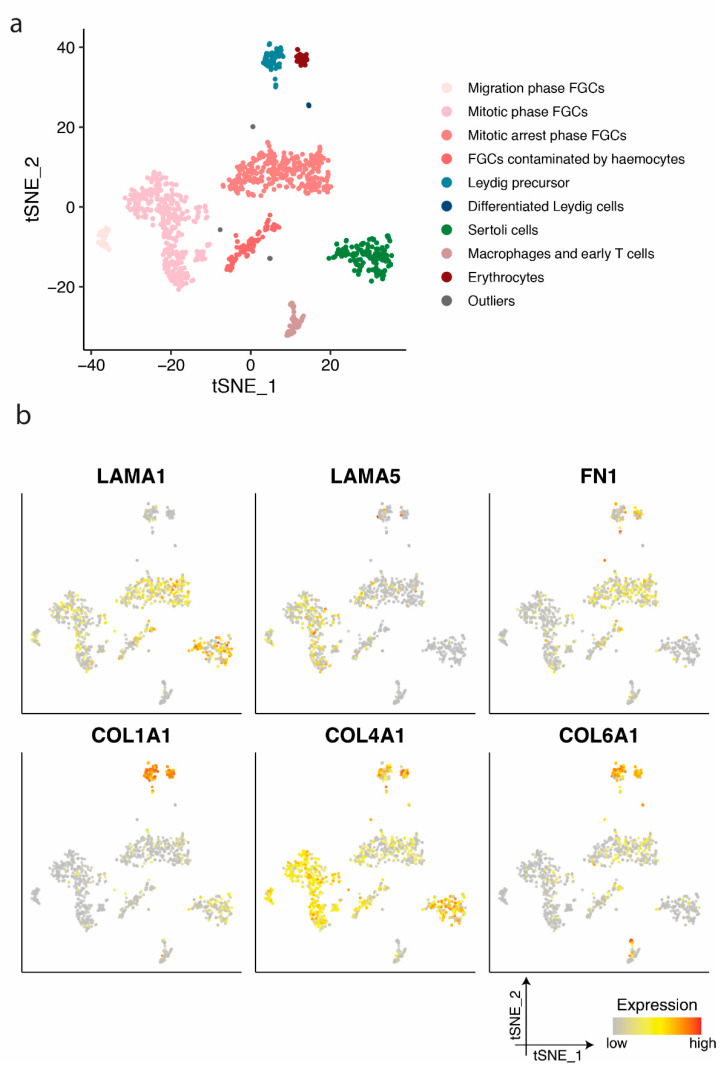
Identification of cell clusters and single-cell transcription profiles of prenatal gonadal cells. (**a**) The t-distributed stochastic neighbour embedding (t-SNE) plot of germ and somatic cells, coloured by the identified cell types. FGC indicates foetal germ cells. (**b**) Single-cell expression profile for extra cellular matrix (ECM) protein coding genes exhibited on the t-SNE plot; gradient of grey, yellow, orange, and red indicates low to high expression.

Based on clusters defined by Li et al. [29], germ and somatic cells from prenatal testes were separated into ten clusters (Figure 2a): Migrating, mitotic, and mitotically arrested foetal germ cells (FGCs), FGCs contaminated by haemocytes, Leydig cell precursors, differentiated Leydig cells, Sertoli cells, macrophages and early T cells, erythrocytes and outliers.

When examining the expression profile of Sertoli cells we noted a high expression of *LAMA1* and *COL4A1* (Figure 2b). *LAMA1* gene expression was also detected in various foetal germ cell phases (Figure 2b), supportive of the LAMA1 protein expression observed in the immature and mature seminiferous membrane enclosing these cell types (Figure 1). *LAMA5* was primarily detected in migratory and mitotic phase foetal germ cells (Figure 2b), whereas no protein expression was detected in the seminiferous basement membrane (Figure 1). The expression of *LAMA5* in mitotic phase germ cells may be a remnant of migratory foetal germ cells. Migratory foetal germ cells demonstrate a similarity to stem cells which rely on *LAMA5*, amongst others, for the maintenance of stemness. Leydig cell precursors exhibited a high expression of *COL1A1*, *COL4A1*, and *COL6A1*. The detection of *COL1A1* supports the protein expression observed in the vasculature and immature seminiferous basement membrane in foetal and prepubertal, as well as the outermost seminiferous membrane layer in the adult (Figure 1). *COL4A1* gene expression was observed mainly in Sertoli, Leydig, and foetal germ cells supporting protein expression in the vasculature, in addition to the immature and mature seminiferous basement membrane. The four germ cell clusters, as defined in a previous study [29], expressed *LAMA1*, *FN1*, and *COL4A1* (Figure 2b). Leydig cell precursors additionally exhibited the expression of *LAMA2* and *LAMA4* (Figure S2). Most cell types demonstrated the expression of *LAMB2* and *LAMC1* (Figure S2).

**Figure 3:**
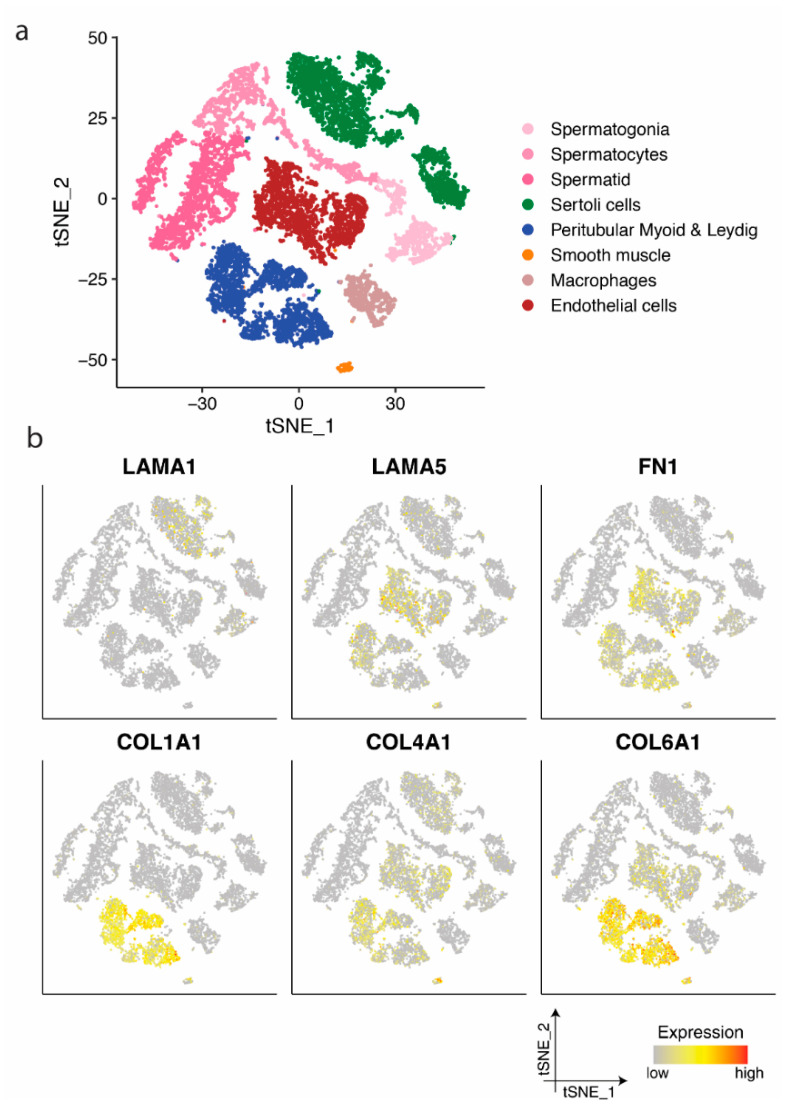
Identification of cell clusters and single-cell transcription profiles of postnatal testicular cells. (**a**) The t-SNE plot of germ and somatic cells, coloured by the identified cell types. (**b**) Single-cell expression profile for ECM protein coding genes exhibited on the t-SNE plot; gradient of grey, yellow, orange, and red indicates low to high expression.

Based on the expression profile, germ and somatic cells from postnatal testes aged 1–25 years were separated into eight clusters (Figure 3a): Spermatogonia, spermatocytes, spermatids, Sertoli cells, peritubular myoid and Leydig cells, smooth muscle cells, macrophage, and endothelial cells, as defined in the previous study [30].

Similar to prenatal samples, the expression of *LAMA1* and *COL4A1* could be observed in prepubertal and adult Sertoli cells, while peritubular myoid and Leydig cells predominantly demonstrated a high expression of *COL1A1*, *COL4A1*, and *COL6A1*, *LAMA2* and *LAMA4*, as well as *FN1* (Figure 3b and Figure S3). Endothelial cells showed mainly the expression of *LAMA5*, *FN1*, and *COL4A1* and *COL6A1* (Figure 3b), while all *LAMA* expressing clusters showed mainly the expression of *LAMB2* and *LAMC1* (Figure S3).

As the analysis of germ cell transcriptomic data has shown the progression of pluripotency factor expressing primordial germ cells (PGC) to a differentiating pre-spermatogonia state throughout prenatal development [29,31], we wanted to investigate potential relationships between the establishment of the BM and differentiation of prenatal germ cell subpopulations.

In order to investigate germ cell subpopulations, the expression of POU5F1 was used as an early PGC marker and DDX4 and MAGE-A4 as markers of progression towards pre-spermatogonia.

**Figure 4:**
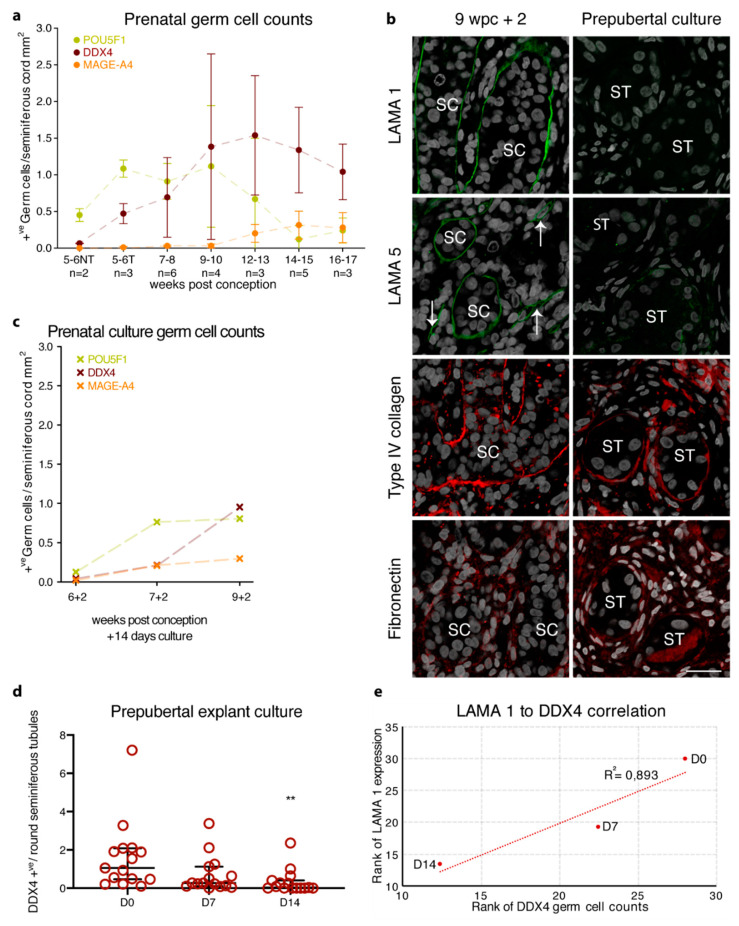
Basement membrane expression of prenatal, prepubertal, and peripubertal tissue cultures in correlation to germ cell loss. (**a**) Prenatal germ cell counts for POU5F1 (green), DDX4 (dark red), and MAGE-A4 (orange) are presented as the mean ±SD of positive germ cells per seminiferous cord mm^2^ from 5 to 17 weeks post conception. NT represents samples with not yet formed seminiferous cords; T represents samples with formed seminiferous cords. (**b**) Following culture for 14 days, the prenatal gonadal tissue shows seminiferous BM expression of LAMA 1 (green staining) and LAMA 5 (green staining), while type IV collagen (red staining) and fibronectin (red staining) show seminiferous BM and interstitial expression. The 14-day pre- and peripubertal testicular cultures show the loss of LAMA 1 expression and weak seminiferous BM expression of LAMA 5 with the seminiferous BM and interstitial expression of type IV collagen and fibronectin. Counterstain with DAPI (grey staining). SC depicts the seminiferous cord, white arrows indicate blood vessels, scale bar: 25 μm (**c**) POU5F1 (green), DDX4 (dark red), and MAGE-A4 (orange) germ cell counts for the prenatal tissue culture are presented as positive germ cells per seminiferous cord mm^2^ for three prenatal culture samples. (**d**) Number of DDX4 positive germ cells per seminiferous tubule cross-section from 16 patients. Median, fifth, and 95th percentile of DDX4 counts are shown for control (D0), D7 and D14 of culture. ** Indicate *p* < 0.01 significance between D0 and D14. (**e**) Spearman’s correlation of DDX4 positive germ cells per seminiferous tubule cross-section in relation to LAMA 1 positive seminiferous tubules for prepubertal explant cultures at D0 (*n* = 13), D7 (*n* = 12), and D14 (*n* = 15).

Germ cell markers were first examined in the first and second trimester prenatal testis development. POU5F1 positive germ cells were present from 5 wpc (0.45 ±0.09 POU5F1^+ve^/mm^2^ of gonad area) in gonads yet to form seminiferous cords. In parallel to the formation of the seminiferous cords, as revealed by the BM expression of LAMA 1, collagens, and fibronectin, POU5F1 positive germ cells increased in number until 9–10 wpc (1.12 ± 0.83 POU5F1^+ve^/mm^2^ seminiferous cord area) then subsequently decreased until 16–17 wpc (0.24 ± 0.17 POU5F1^+ve^/mm^2^ seminiferous cord area) (Figure 4a and Figure S4, Tables S1 and S2). DDX4 positive germ cells were detected with the onset of seminiferous cord formation around 6 wpc and increased in quantity until 12–13 wpc (1.54 ± 0.81 DDX4^+ve^/mm^2^ seminiferous cord area) with a minor decrease by 16–17 wpc (1.04 ± 0.38 DDX4^+ve^/mm^2^ seminiferous cord area) (Figure 4a and Figure S4, Tables S1 and S2).

MAGE-A4 positive germ cells were observed at the onset of seminiferous cord formation around 6 wpc (0.01 ± 0.02 MAGE A4^+ve^/mm^2^ seminiferous cord area) and increased slightly in quantity until 16–17 wpc (0.28 ± 0.21 MAGE-A4^+ve^/mm^2^ seminiferous cord area) (Figure 4a and Figure S4, Tables S1 and S2). A comparison of the germ cell quantity per tubular cord area of the first trimester (5–10 wpc with established seminiferous cords) to the second trimester (12–17 wpc) samples revealed a statistically significant decrease in POU5F1 (1.01 ± 0.47 vs. 0.30 ± 0.46; *p* < 0.001) with a corresponding significant increase in DDX4 (0.85 ± 0.84 vs. 1.31 ± 0.58; *p* < 0.05) and MAGE-A4 (0.03 ± 0.02 vs. 0.27 ± 0.17; *p* < 0.001).

As the non-pathological prepubertal testicular tissue is difficult to obtain, testicular biopsy samples from 16 prepubertal boys (1.6–13.4 years of age) (11 not exposed to any therapy and five exposed to non-alkylating agents prior to biopsy) enrolled in the NORDFERTIL fertility preservation initiative were used for the BM evaluation. A previous study from our group, which included 12 of the 16 patients used in the current study, demonstrated equivalent germ cell counts for non-treated and non-alkylator treated patients compared to a reported reference range and biobank controls [32]. This allowed us to pool both treatment groups and consider them as reference samples in regard to germ cell counts.

Similar to prenatal samples, prepubertal samples showed the expression of type IV collagen, fibronectin, and LAMA 1 in the seminiferous BM. Type VI collagen and fibronectin were additionally expressed in the interstitium and forming peritubular compartment (Figure 1 and Table S3). No expression of LAMA 2, 3, 4, and 5 could be observed in the seminiferous BM, while LAMA 5 showed a vascular expression similar to prenatal samples (Figure 1).

As the analysis of germ cell transcriptomic data has revealed the presence of multiple germ cell populations with distinct expression patterns [33], but a common expression of DDX4 in all prepubertal germ cell populations, we focused the prepubertal germ cell evaluation on the expression of DDX4 in order to obtain an overview of the whole germ cell population. DDX4 positive germ cells of both non-treated and non-alkylator treated prepubertal testicular tissue showed the expression of 1.54 ± 1.75 DDX4^+ve^/round seminiferous tubule (Figure 4d and Figure S4).

For the investigation of adult BM composition, biobank samples without the underlying pathologies were analysed. In contrast to the prenatal seminiferous BM, the adult testis displayed a multi-layered structure, with an innermost basal lamina, a middle collagenous layer, and an outermost peritubular layer, as previously described [34]. Type I, IV, and VI collagens, fibronectin, and LAMA 1 were localised in distinct layers of the BM (Figure 1). Type I, IV, and VI collagens, as well as fibronectin showed expression in the collagenous and peritubular layer of the seminiferous BM, while type IV collagen showed further expression in the basal lamina of seminiferous BM (Figure 1 and Figure S1). LAMA 1 showed expression solely in the basal lamina of the seminiferous BM, while LAMA 5 was expressed in the vasculature (Figure 1). In contrast to prenatal and prepubertal samples, adult testicular samples demonstrated the expression of LAMA 2 and 4 in the vasculature and the peritubular layer of the seminiferous BM (Figure S1). The cytoplasmic expression of LAMA 2, based on morphology and location, could further be observed in early meiotic spermatocytes, while LAMA 4 could be detected as a punctuate staining in spermatocytes of the seminiferous tubules (Figure S1). No LAMA 3 was observed in the adult samples (Figure S1).

Overall, we have described the conserved expression of LAMA 1, type I, IV, and VI collagens, and fibronectin upon seminiferous cord formation up until adulthood with the onset of discrete expression patterns upon the formation of the multi-layered adult seminiferous BM.

### 2.2. Basement Membrane Disruption upon Testicular Culture Correlates with Germ Cell Loss

The impaired maturation of the somatic environment is speculated to underlie the gradual loss of germ cells observed during the culture of testis tissue obtained for fertility preservation [2,3,4]. To examine whether fertility preservation approaches such as the testicular organ culture disrupt the BM composition, and with this contribute to germ cell loss, the testicular organ culture using an air-liquid interface was performed for 14 days and the BM composition was evaluated. Since the establishment of the seminiferous BM is initiated prenatally at which time germ cells have not yet migrated towards the seminiferous BM, and migration towards the seminiferous BM occurs postnatally, cultures were performed with both prenatal and prepubertal testicular tissues.

The expression profile of three (6, 7, and 9 wpc) 14-day cultured prenatal male gonads showed a similar profile to age-matched in vivo controls with LAMA 1, type I, IV, and VI collagens, and fibronectin expression in the seminiferous BM (Figure 4b). Surprisingly, the LAMA 5 expression could not only be observed in the vasculature but also in the seminiferous BM upon culture (Figure 4b). Despite the atypical seminiferous BM expression of LAMA 5, the expression of all three germ cell markers could be observed after 14 days of culture with similar quantities compared to the age-matched in vivo samples (Figure 4c and Table S1).

Cultured prepubertal samples showed similar seminiferous BM expression patterns of type IV collagen and fibronectin compared to the culture controls (Figure 4b). Evaluation of caspase 3 (CASP3; marker for apoptotic cells) and KI67 (marker for proliferating cells) protein expression showed a slight increase for both markers after 7 and 14 days in culture compared to the non-cultured tissue (Figure S5). While CASP3 positive cells were primarily located in the interstitial compartment of the tissue, KI67 positive cells were located in both interstitial and intratubular compartments. Interestingly, four out of 16 samples showed a weak seminiferous LAMA 5 expression (Figure 4b). Surprisingly, cultured prepubertal samples showed a significant decrease in seminiferous LAMA 1 expression compared to culture controls (D0 78.4% ± 33.5 vs. D7 36.9% ± 35.9 and D14 15.5% ± 28.5 LAMA 1^+ve^ seminiferous tubules; *p* < 0.001) with a complete loss of LAMA 1 at 14 days of culture in nine out of 16 samples (Figure 4b and Table S3). Evaluation of DDX4 showed a significant loss of DDX4 positive germ cells by 14 days of culture (D0 1.54 ± 1.75 vs. D14 0.31 ± 0.62 DDX4^+ve^/round seminiferous tubules; *p* < 0.01) (Figure 4d and Figure S4, Table S3), which was shown to correlate with the loss of seminiferous LAMA 1 expression (Figure 4e).

## 3. Discussion

BMs are an important part of various stem cell niches, supporting cell signalling, proliferation, polarization, and migration.

**Figure 5:**
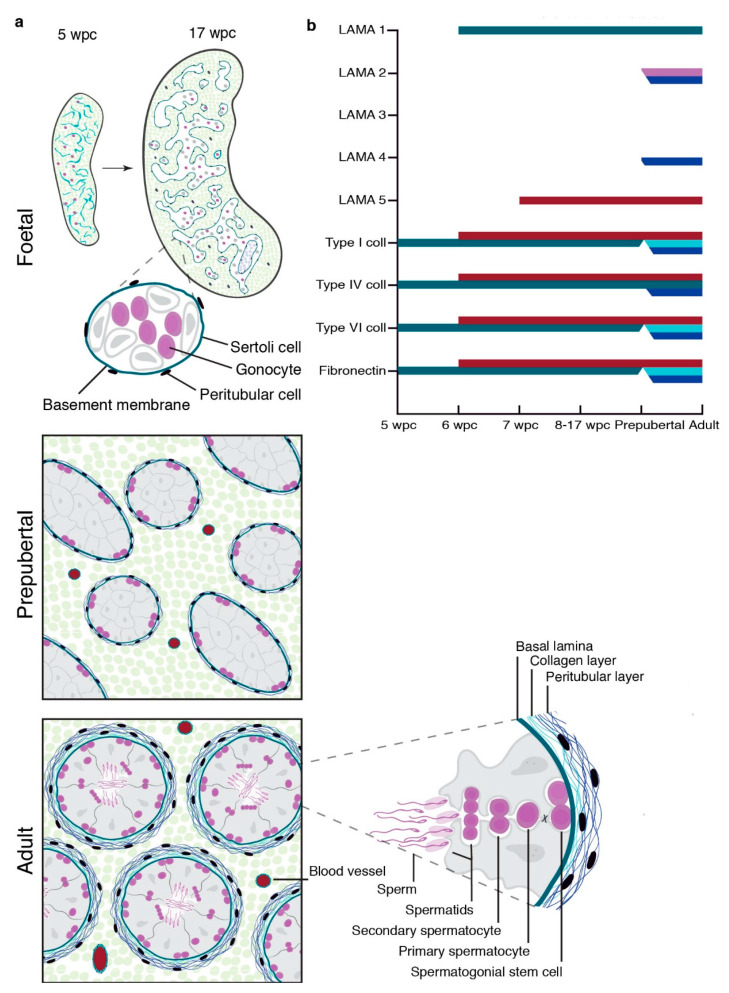
Schematic depiction of testicular LAMA, fibronectin, and collagen expression during gonadal development. (**a**) Establishment of a single-layered seminiferous basement membrane from 5 to 17 wpc, prepubertal, and multi-layered structure of adult seminiferous tubules. (**b**) Onset of LAMA 1, 2, 3, 4, and 5 as well as type I, IV, and VI collagens and fibronectin expression in prenatal first and second trimester gonads, as well as their expression pattern in prepubertal and adult testicular tissue. Colour coding: Basal lamina (green), collagenous BM layer (light blue), peritubular BM layer (dark blue), spermatocytes (pink), and vasculature (red).

Here, we have demonstrated that LAMA 1, collagen type I, IV, and VI, and fibronectin are the primary components of the pre- and postnatal BM in humans, with discrete expression patterns within the multi-layered adult seminiferous BM (Figure 5a,b). Further, we revealed a loss of seminiferous LAMA 1 expression following the testicular organ culture as a fertility preservation approach for the prepubertal testicular tissue, which correlated with a progressive depletion of germ cells.

In contrast to mice, that show the expression of LAMA 1, 2, 4, and 5 in the seminiferous BM [18], we reveal the composition of the human seminiferous BM to be more conserved, describing LAMA 1 as the sole LAMA chain located in the seminiferous BM in prenatal and prepubertal gonads. In adult human tissue, it is expressed at the innermost layer, in direct contact with the Sertoli cells and spermatogonia, while LAMA 2 and 4 are primarily expressed in the peritubular compartment, spatially separated from the spermatogonia and Sertoli cells.

Pre- and postnatal single-cell RNA sequencing gene expression profiles give additional insights into the discrete testicular protein expression pattern in the adult compared to prenatal. In the final structured adult testis, Sertoli and germ cells enclosed in the seminiferous compartment likely contribute to the production of the innermost seminiferous BM (LAMA 1 and type 4 collagen), while Leydig and peritubular cells located in the interstitial space contribute to the production of the outermost seminiferous BM (type I and VI collagen, fibronectin, LAMA 2 and 4). Although, we could not show clusters and gene expression profiles for prenatal peritubular cells, our results confirmed the previous mass spectrometry analysis of cultured adult human peritubular cells [10], demonstrating gene expression profiles, as well as protein expression of type I, IV, and VI collagens, LAMA 2 and 4 in postnatal testes, with the addition of fibronectin in the peritubular layer of the adult seminiferous BM. Such expression profiles suggest that peritubular cells contribute ECM components to both the interstitial ECM, as well as the outermost seminiferous BM, in the adult testis.

Interestingly, LAMA 2 was further expressed in the cytoplasm of early to meiotic spermatocytes of adult testicular tissue with an established blood-testis-barrier. In vitro rodent studies involving the knockdown of LAMA 2 expression in Sertoli cell cultures have resulted in the disruption of tight junction formation, while LAMA 2 knock-out leads to impaired spermatogenesis, suggesting a regulatory role in the regulation of the blood-testis-barrier [9,19,20]. Given that we were only able to localise LAMA 2 and 4 protein expression in the adult seminiferous BM and meiotic spermatocytes, with no such expression observed in prenatal or prepubertal samples, we suggest a role in progressive spermatogenesis. Nevertheless, further investigations will be required to evaluate whether the involvement of LAMA 2 in the regulation of the blood-testis-barrier and spermatogenesis is comparable to the role in rodents.

As germ cell loss is commonly observed in human testicular culture models, limiting the progress of spermatogenesis in vitro, we sought to examine the relationship between the BM composition and germ cell population. We first determined reference values for the germ cell populations from gonocyte (POU5F1) to (pre)spermatogonia (MAGE-A4) during prenatal gonad development. In agreement with the previous studies, POU5F1 positive germ cells are already present once primordial germ cells colonise the primitive genital ridges (5 wpc) and maintain expression until 17 wpc [35,36]. In contrast to previous studies, we could observe DDX4 and MAGE-A4 positive germ cells from the establishment of LAMA 1 expressing seminiferous cords which increased in quantity until 17 wpc. Although we could observe a progressive establishment of distinct germ cell populations based on the expression pattern, it is not clear whether the establishment of different germ cell populations is correlated to the presence of LAMA 1 or due to the progression of Sertoli cell maturation.

While protein expression patterns in 14-day cultured prenatal gonads were comparable to age-matched in vivo controls, the appearance of LAMA 5 expression was observed in the seminiferous BM. Despite suggesting BM changes due to suboptimal culture conditions, the onset of LAMA 5 expression did not correlate with a depletion of germ cells. The persistence of LAMA 1 expression upon the culture of prenatal samples and the luminal location of germ cells in prenatal samples could underlie the minimal germ cell disruption observed.

Interestingly, the significant loss of seminiferous LAMA 1 expression in prepubertal testicular tissue samples after 14 days of culture correlated with a significant germ cell loss. Although, cultured prepubertal tissues were viable, a significant loss in DDX4 positive germ cells could be observed between D0 controls taken at the time of biopsy and day 14 of culture, in agreement with the previous studies [2,3]. Since the rapid germ cell loss was correlated only with the loss of LAMA 1 expression, and no other BM components, or a dramatic increase in apoptosis during culture, we suggest LAMA 1 to be crucial in the survival and maintenance of germ cells which have already undergone translocation to the seminiferous BM. This finding is supported by a recent study from Murdock et al. showing human testicular ECM extracts to be important for spermatogonia stem cell maintenance [37]. The loss of ECM components has a negative effect on spermatogonia maintenance, as well as progression and reduced spermatogonia maintenance on human and mouse fibroblast laminin extracts, which comprise LAMA 5 and 3 is observed [37]. Furthermore, in the same study, Murdock et al. showed that despite the testicular ECM extract, all used culture conditions resulted in a gradual decline in spermatogonia during 2 weeks of culture, highlighting the importance of maintaining an intact testicular environment, including the ECM as well as all cell types, to be crucial for spermatogonia maintenance and progression. Nevertheless, further studies will be required to evaluate whether the observed germ cell loss is based on a lack of proper adhesion molecules, disruption of intracellular signalling, or both. Given that the loss of LAMA 1 was only observed in prepubertal samples, further investigation should focus on the differences between Sertoli cell subpopulations in prenatal and prepubertal tissues.

In light of our results, further studies will be required to optimize culture conditions to preserve the laminin composition of the BM and subsequent germ cell maintenance.

In conclusion, our study has demonstrated an important role for LAMA 1 in the maintenance and support of the SSC niche. Our studies have relevance for the evaluation of testicular pathologies, as well as for the improvement of in vitro germ cell derivation, differentiation, and maintenance.

## 4. Materials and Methods

### 4.1. Ethical Permission

The human first trimester tissue was collected after elective surgical terminations with a maternal written informed consent. Midwives were responsible for informing patients (over 18 years of age and Swedish speaking). The Regional Human Ethics Committee, Stockholm, Sweden, approved the collection (Dnr 2007/1477-31 with complementary permissions 2011/1101-32 and 2013/564-32. The ethical approval to perform the gonadal studies: Dnr 2013/457-31/4).

Ethical approval for use of human adult testis tissues was obtained from the East of Scotland Research Ethics Committee (reference: 15/ES/0094). Ethical approval for use of human prenatal testis tissues was obtained from the South East Scotland Research Ethics Committee (reference: LREC08/S1101/1) and NRES committee North East–Newcastle and North Tyneside 1 (reference: 18/NE/0290). All women gave a written informed consent. Ethical approval for use of prepubertal testicular tissues was obtained from the ethics Board of Karolinska Institutet and the Regional Ethics Board in Stockholm (Dnr 2013-2129-31-3), the National Ethics Board of Iceland, Reykjavik (VSN 15-002), and the Ethics Board of the University of Helsinki (426/13/03/03/2015).

### 4.2. Sample Collection and Preparation

The first human male trimester gonadal tissue, ranging from 5–10 wpc (*n* = 14) was obtained from elective surgical abortions at the Karolinska University Hospital. The wpc age of the tissue was determined by the examination of anatomical landmarks such as the nervous system, limb, eye, and gonadal development according to the atlas of England [38]. As the collected residual tissue was not intact after a surgical routine procedure, the crown rump length (CRL) or neck rump length (NRL) was often not possible to measure. The accuracy of staging may vary to a maximum of ±0.5 weeks.

Within 1 to 2 h after surgery, the tissue was dissected in a physiological sodium chloride solution under sterile conditions. Thereafter, the gonad was transferred to Knock-out Dulbecco’s Modified Eagle Medium (Invitrogen, Carlsbad, CA, USA). Gonads were fixed in 4% paraformaldehyde and paraffin embedded, while the additional tissue was used for sex determination. To increase the number and age-range of tissue samples included in this study, the human male first and second trimester gonads, ranging from 9–17 wpc (first trimester *n* = 1, second trimester *n* = 12), were obtained following an elective termination of pregnancy at the University of Edinburgh. No terminations were due to embryonic or foetal abnormalities. Tissues were fixed in 4% paraformaldehyde and paraffin embedded.

Prepubertal boys in Sweden, Finland, and Iceland, between 1.6 and 13.4 years of age, who were facing treatments associated with a very high risk of infertility (allogeneic or autologous HSCT or testicular radiotherapy), were offered the experimental procedure of testicular cryopreservation. Patients underwent unilateral open testicular biopsy where less than 20% of the testicular volume was sampled. Two thirds were cryopreserved for clinical fertility preservation and the remaining third was pseudonymized and transported to the NORDFERTIL research laboratory at Karolinska Institutet. Exclusion criteria for testicular biopsy were pre-existing spermatogenesis (testicular volumes >10 mL by orchidometer) and a high bleeding or infection risk. The parents and, where appropriate, the patient received verbal and written information about the research project and gave their written informed consent. Testicular biopsy samples from 16 boys (11 not exposed to any therapy and five exposed to non-alkylating agents prior to biopsy), 12 patients of which were included in a previous study [32], were fixed in 4% formalin, in phosphate buffered saline, and embedded in paraffin. As samples from both non-treated and non-alkylator treated patients have germ cell counts equivalent to a reported reference range and biobank controls, both groups could be pooled and considered as reference samples. Three adult control testes from biobank samples without underlying pathologies (with respect to germ cell composition) were obtained at the University of Edinburgh and fixed in 10% neutral-buffered formalin and embedded in paraffin.

### 4.3. Testicular Explant Tissue Cultures

First trimester testicular tissue samples ranging from 6–9 wpc, as well as pre- and peripubertal testicular tissue samples ranging from 1.6 to 13.4 years (mean age: 7.1 +/− 4.2 years) from the NORDFERTIL research cohort were cultured using a modified testicular explant culture system established by Sato et al. [5]. In brief, whole first trimester gonads were cut into three equal pieces, while the testicular biopsy samples from prepubertal boys were cut into pieces of approximately 1 mm^3^. Each of the tissue fragments were cultured on top of a 0.35% agarose block in an air-liquid interface. To prepare the agarose blocks, autoclaved 0.7% SeaKem^®^ LE agarose (50004, Lonza, Basel, Switzerland) was mixed 1:1 with NutriStem (05-100-1, Biological Industries, Cromwell, CT, USA) with 1% penicillin and streptomycin (Pen/Strep, 15140-122, Invitrogen) and poured into 24-well plates, 1 mL per well. After solidification, blocks were cut out using the proximal end of 1000 µL pipette tips (diameter of 0.8 mm). The tissue pieces were cultured in NutriStem with a 10% knock-out serum replacement (KO-SR, 10828-028, Invitrogen), 1% penicillin-streptomycin, and 10^−7^ M melatonin (M5250, Sigma-Aldrich, Darmstadt, Germany) for 14 days with media change at day 7 of culture. Cultures were performed at 37 °C for prenatal and or 35 °C for prepubertal tissue, respectively. At day 14 of culture, tissues were fixed in 4% paraformaldehyde and embedded in paraffin.

### 4.4. Immunofluorescence Staining

Paraffin embedded tissue samples were cut into 5 µm thin sections, de-paraffinized in xylene, and rehydrated through a graded alcohol series. Sections were subjected to a heat-mediated antigen retrieval in either 10 mM sodium citrate (Sigma-Aldrich) or 10 mM Tris base (Sigma-Aldrich) and a 1 mM EDTA (Merck-Millipore, Darmstadt, Germany) solution with 0.05% Tween 20 (Merck-Millipore) at pH 9.

Sections were blocked in Tris-buffered saline (TBS) consisting of 150 mM NaCl (Sigma-Aldrich) and 50 mM of Tris base at a pH 7.6 containing 10% donkey serum (125558, Jackson Immuno Research, Cambridgeshire, UK) and 1% bovine serum albumin (001-000-162, Jackson Immuno Research). The primary antibody incubation was performed overnight at 4 °C in a 1:2 TBS diluted blocking buffer (for antibody information see Supplementary Table S4). Negative controls were performed with mouse and rabbit IgGs (see Table S4 and Figure S6).

After washing the slides in a TBS buffer, the secondary antibody incubation was performed at room temperature for 1 h in a 1:2 TBS diluted blocking buffer with a secondary antibody: Donkey anti rabbit Cy3 (711-166-152, Jackson Immuno Research) and donkey anti mouse AF488 (715-546-150, Jackson Immuno Research). Slides were counter-stained with DAPI (135-1303, Bio-Rad, Hercules, CA, USA) at a concentration of 1 mg/mL for 10 min and mounted with a Pro Long Gold antifade reagent (P36931, Thermo Fisher Scientific, Waltham, MA, USA).

### 4.5. Imaging

Imaging of whole tissue sections was performed with a Zeiss LMS700 confocal microscope (Zeiss, Jena, Germany) as tile scans and a positive signal were evaluated in comparison to isotope controls.

### 4.6. Cell Quantification and Statistical Analysis

Quantification of POU5F1, DDX4, and MAGE-A4 positive cells, as well as counting of collagen IV positive blood vessels and evaluation of tubular cord area of the prenatal tissue, were performed using one tissue section of the whole gonad for each condition. Results are shown as the mean ± standard deviation (SD). The Kruskal-Wallis one way analysis of variance on ranks was performed to confirm the statistical significance between the first and second trimester gonadal counting. Analyses were performed with the SigmaPlot 13.0 software (Systat Software Inc., Chicago, IL, USA).

Quantification of DDX4 positive germ cells, as well as the percentage of LAMA 1 positive seminiferous tubules in pre- and peripubertal testes, were performed on at least one tissue section of one testicular explant culture or culture control sample. A mean of 39 and 20 seminiferous tubules per patient were evaluated for DDX4 and LAMA 1, respectively. Germ cells were evaluated from round seminiferous tubules (RT). Results are shown as mean ±SD. One-way ANOVA or one-way ANOVA on ranks was performed to confirm the statistical significance between pre- and peripubertal culture and culture-controls. The analyses were performed with the SigmaPlot 13.0 software (Systat Software Inc.).

For the CASP3 and KI67 analysis, positive nuclei per DAPI nuclei were counted with the ImageJ Particle Analyzer. DAPI nuclei were counted after background subtraction (rolling ball radius 50), gaussian blur application (sigma = 3), and image conversion (Otsu threshold function). CASP3 and KI67 positive nuclei were counted after background subtraction (rolling ball radius 50), gaussian blur (sigma = 3), and image conversion (Yen threshold function). The tissue sample area was determined from DAPI staining using a Gaussian blur of 10 and MinError threshold function. Quadrants of images were counted separately as technical replicates.

In R, counted nuclei were pre-filtered for nuclei larger than 5 µm^2^ (DAPI-positive nuclei) or larger than 15 µm^2^ (CASP3 and KI67). To exclude the quadrants with a background signal amplification from the absence of positive staining, we excluded the quadrants with calculated staining of more than 50% KI67 or CASP3 per tissue area. CASP3 or KI67 positive nuclei per DAPI positive nuclei were determined for each quadrant. The mean number of CASP3 or KI67 positive cells was calculated over quadrants to evaluate the technical replication. The statistical difference between samples was calculated using the Wilcoxon rank sum test on biological replicates.

### 4.7. Single-Cell RNA-Sequencing Data Processing

The single-cell RNA-sequencing data of human FGCs and their gonadal niche cells, published by Li et al., were downloaded from https://github.com/zorrodong/germcell [29]. Only male samples (1079 of 2210 cells) were selected, and the expression values on transcripts per million (TPM) were normalized by log2 (TPM/10 + 1). A total of 17,633 genes with expression levels higher than 1 that were expressed over 10 single cell samples were analysed using the R (version 3.5.1) package Seurat (version 2.3.4) [39]. The t-distributed stochastic neighbour embedding (t-SNE) dimensionality reduction was carried out following the protocol of Li et al. [29], in which male and female samples were analysed collectively. First, 360 highly variable genes with an average expression greater than 2 and dispersion more than 2 were selected using the “FindVariableGenes” function and the scaled data were applied for the principal component analysis (PCA). Then, significant principal components (PCs) were determined by the jackstraw procedure with 1000 replicates. Here, PC 1–12 were selected as significant (*p* < 1e-5), and t-SNE was performed using these PCs with the “RunTSNE” function. Cells were clustered using the “DBClustDimension” function with parameters G.use = 3. Finally, the resulting clusters were compared, and annotated based on the classification by Li et al. [29]. Even though those cells in the endothelial cell cluster were classified as outliers or into other clusters, 1035 of 1072 cells (96.5%) were consistently clustered. The single-cell RNA-sequencing data of human postnatal testes were processed as described elsewhere [30]. Gene expression overlays onto the t-SNE plots were performed using the “FeaturePlot” function modified with ggplot2 (version 3.1.0).

## 5. Conclusions

This study highlights the significance of laminin alpha 1 in the spermatogonial stem cell niche and its importance for germ cell maintenance. In light of our results, we propose maintaining an intact basement membrane as part of a functional spermatogonial stem cell microenvironment as a key factor to achieve in vitro maturation of immature human germ cells, and therefore, provide an important first step towards developing a clinical tool for fertility preservation in boys.

## Data Availability

Data is contained within the article or Supplementary Material. The data presented in this study are available in Supplementary Materials.

## Supplementary Materials

The following are available online at https://www.mdpi.com/2073-4409/10/2/241/s1, Figure S1: Extended basement membrane expression of prenatal gonadal and adult testicular tissue.Figure S2: Identification of cell clusters and single-cell transcription profiles of prenatal gonadal cells (4–25 wpc). Single-cell expression profile for ECM proteins exhibited on t-SNE plot; gradient of grey, yellow, orange and red indicates low to high expression. Figure S3: Identification of cell clusters and single-cell transcription profiles of postnatal testicular cells (1–25 years of age). Single-cell expression profile for ECM proteins exhibited on t-SNE plot;gradient of grey, yellow, orange and red indicates low to high expression. Figure S4: Expression of POU5F1 (red staining), DDX4 (green staining) and MAGE-A4 (green staining) positive germ cells can be observed in seminiferous cords of controls and prenatal gonads after 14 days of culture. Prenatal sample *n* = 27, prenatal culture samples *n* = 3. DDX4 positive germ cells can be observed in seminiferous tubules of prepubertal control and 14-day cultured tissue. Prepubertal sample *n* = 16, prepubertal culture samples *n* = 16. Counterstain with DAPI (grey staining). SC depicts the seminiferous cord, scale bar: 25 μm, Figure S5: Increased expression of CASP3 and KI67 positive cells over 14 days in explant tissue culture of prepubertal testis samples (1.56 to 13.39 years of age). (a) Percentage of KI67 stained prenatal samples at day 0 *n*=7, at day 7 *n* = 11 and at day 14 *n* = 10 as well as percentage of CASP3 stained prenatal samples at day 0 *n* = 6, at day 7 *n* = 9 and at day 14 *n* = 8. ** indicate p < 0.01 significance between day 06 and day 7, or day 0 and day 14. (b) Representative images of KI67 (red staining) and CASP3 (red staining) expression in prepubertal testicular tissue samples at day 0 (D0), day 7 (D7) and day 14 (D14) is indicated in selected cells with arrows. Counterstain showing with DAPI (grey staining). Scale bars: 100 μm, zoom scale bars: 10 μm Figure S6: Negative IgG control for uncultured and cultured prenatal gonadal tissue. Counterstain with DAPI (grey staining). SC depicts the seminiferous cords, scale bar: 25 μm Table S1: Immunohistological evaluation of grouped prenatal male gonads, Table S2: Samples and immunohistological evaluation of individual prenatal gonadal tissue Table S3: Immunohistological evaluation of individual postnatal control and cultured gonads Table S4: Primary and secondary antibodies.
