# Supplementary material for: Spermatogonia Loss Correlates with LAMA 1 Expression in Human Prepubertal Testes Stored for Fertility Preservation
Source: Cells. 2021 Jan 27;10(2):241. doi: 10.3390/cells10020241 (PMC7911157; doi:10.3390/cells10020241)
Supplement: Supplementary file 1 [file cells-10-00241-s001.pdf]

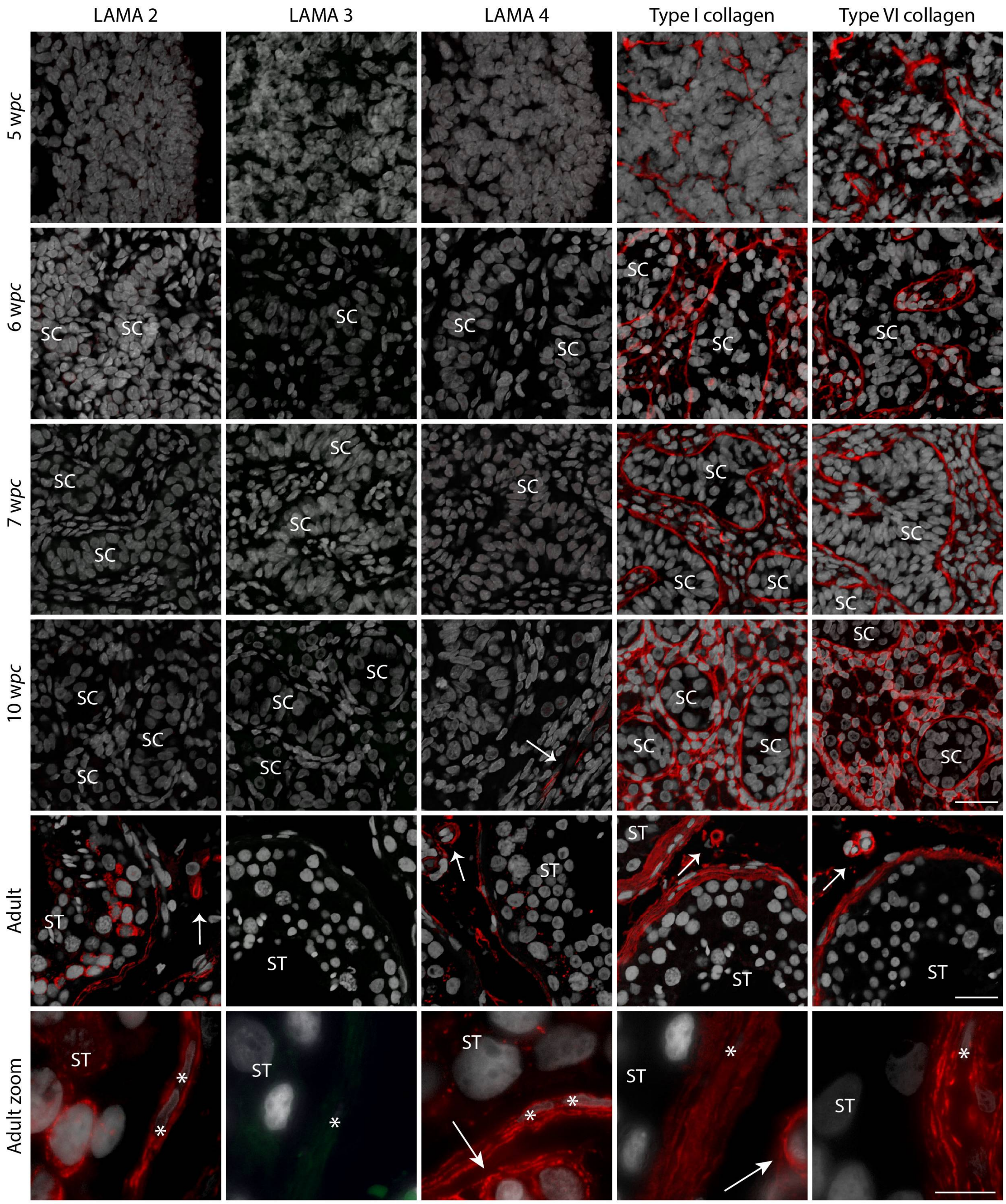

Figure S1: Extended basement membrane expression of prenatal gonadal and adult testicular tissue.

No expression of LAMA 2 (red staining), 3 (green staining) and 4 (red staining) can be observed in prenatal gonadal tissue. Type I (red staining) and VI (red staining) collagen expression can be observed from 5 wpc as net like structure and in the seminiferous BM and interstitum from 6 wpc. LAMA 2 and 4 expression can be observed in the peritubular BM layer, meiotic germ cells and the

vasculature, while no LAMA 3 expression can be observed in adult testicular tissue. Type I and VI collagen can be observed in peritubular and collagenous layer of the seminiferous BM and the vasculature. Prenatal sample n=27; adult sample n=3. Counterstain with DAPI (grey staining). SC depicts the seminiferous cord, ST depicts seminiferous tubules, white arrows indicate blood vessels, blue arrows show innermost BM protrusions between tubular cells, * indicate peritubular cells, scale bar: 25µm, zoom scale bar: 10µm

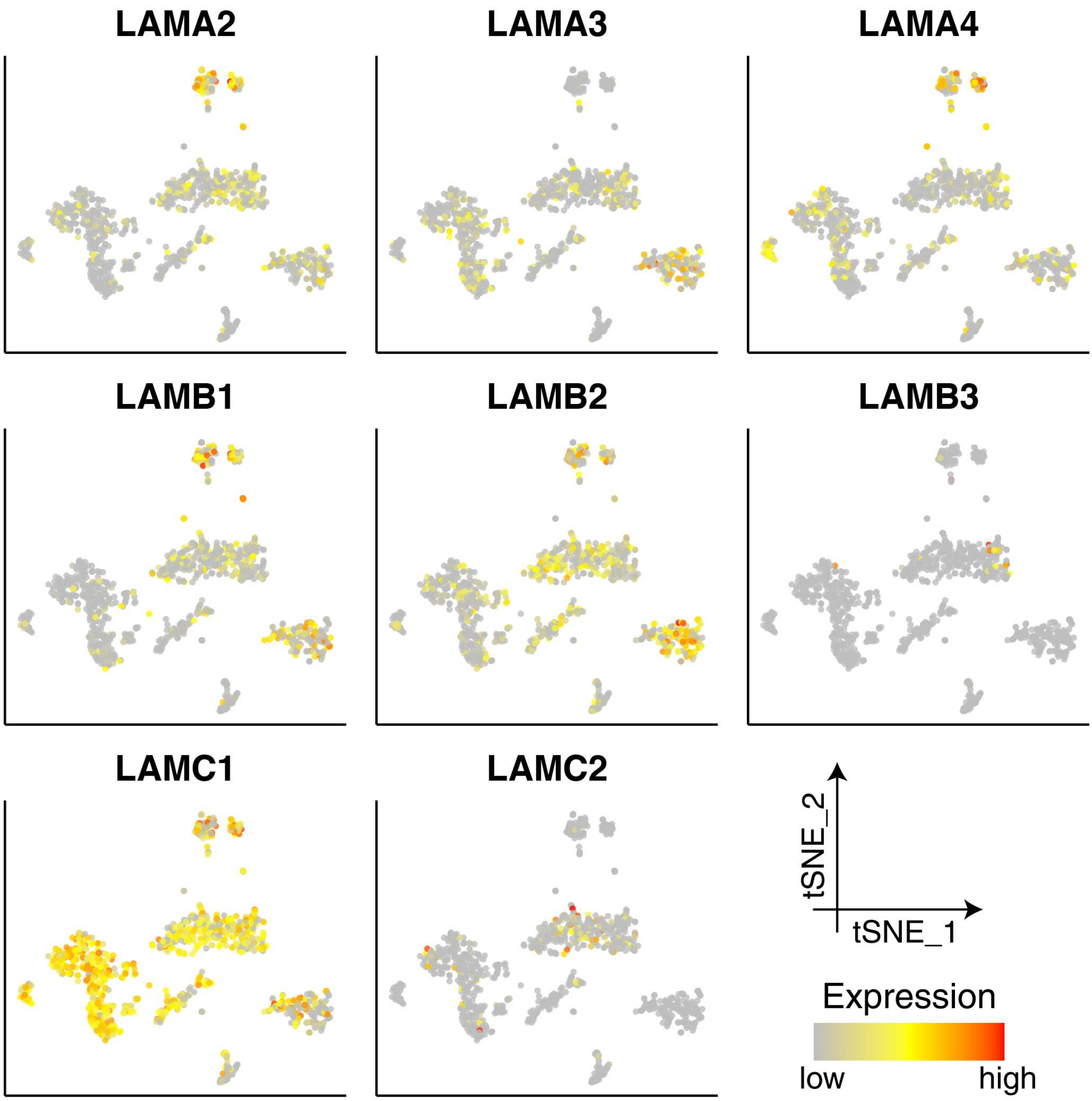

Figure S2: Identification of cell clusters and single-cell transcription profiles of prenatal gonadal cells (4-25 wpc). Single-cell expression profile for ECM proteins exhibited on t-SNE plot; gradient of grey, yellow, orange and red indicates low to high expression.

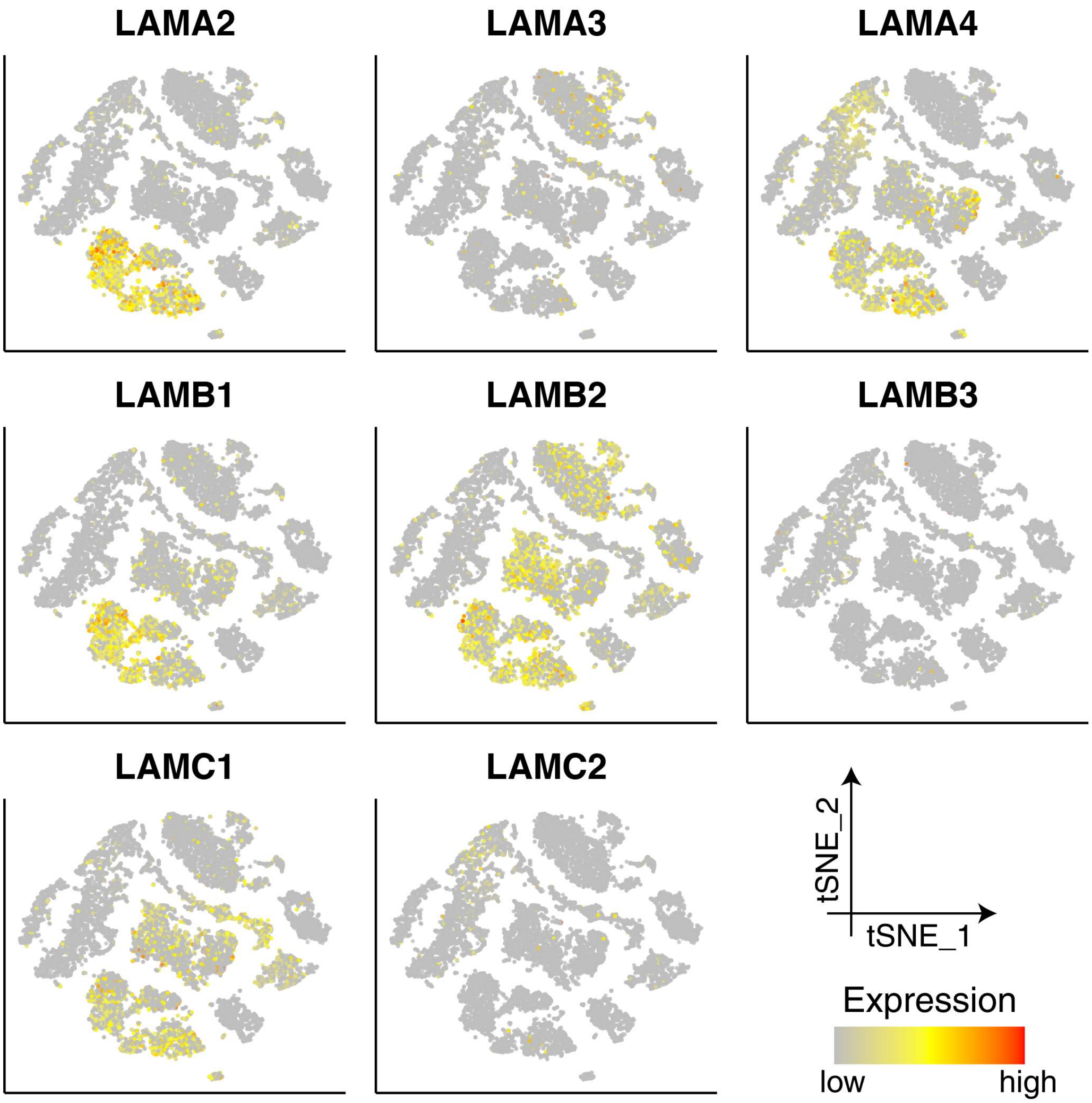

Figure S3: Identification of cell clusters and single-cell transcription profiles of postnatal testicular cells (1-25 years of age). Single-cell expression profile for ECM proteins exhibited on t-SNE plot; gradient of grey, yellow, orange and red indicates low to high expression.

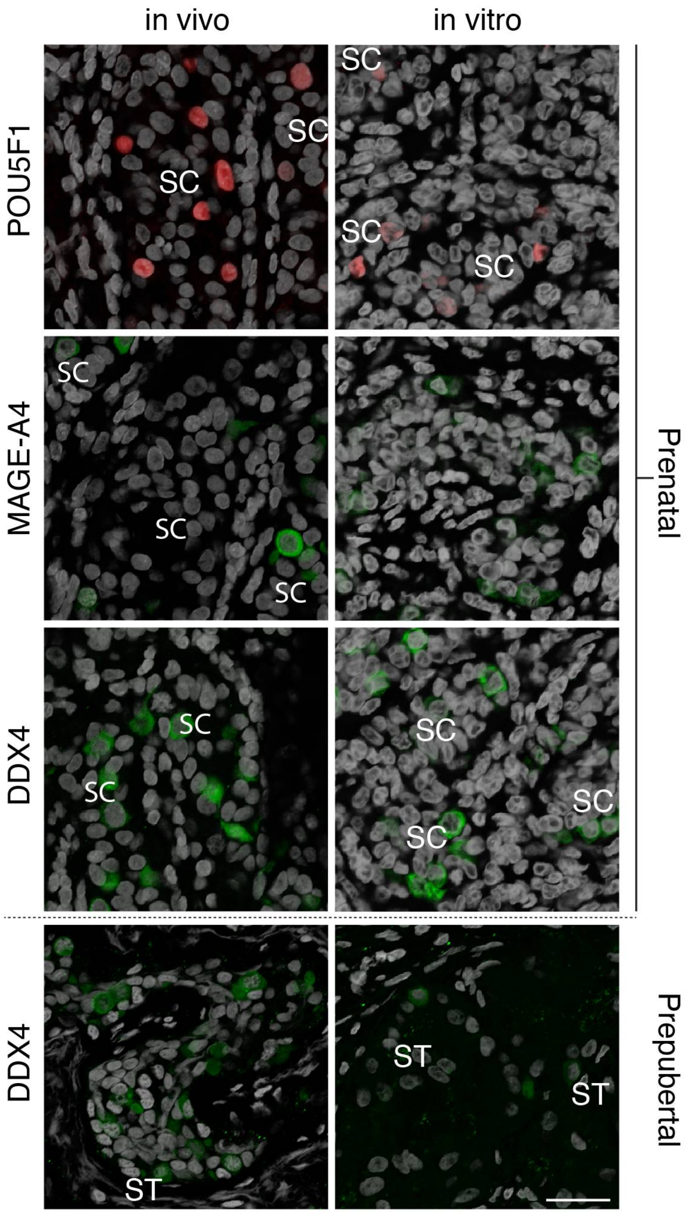

Figure S4: Expression of POU5F1 (red staining), DDX4 (green staining) and MAGE-A4 (green staining) positive germ cells can be observed in seminiferous cords of controls and prenatal gonads after 14 days of culture. Prenatal sample n=27, prenatal culture samples n=3. DDX4 positive germ cells can be observed in seminiferous tubules of prepubertal control and 14-day cultured tissue. Prepubertal sample n=16, prepubertal culture samples n=16. Counterstain with DAPI (grey staining). SC depicts the seminiferous cord, scale bar: 25 μm

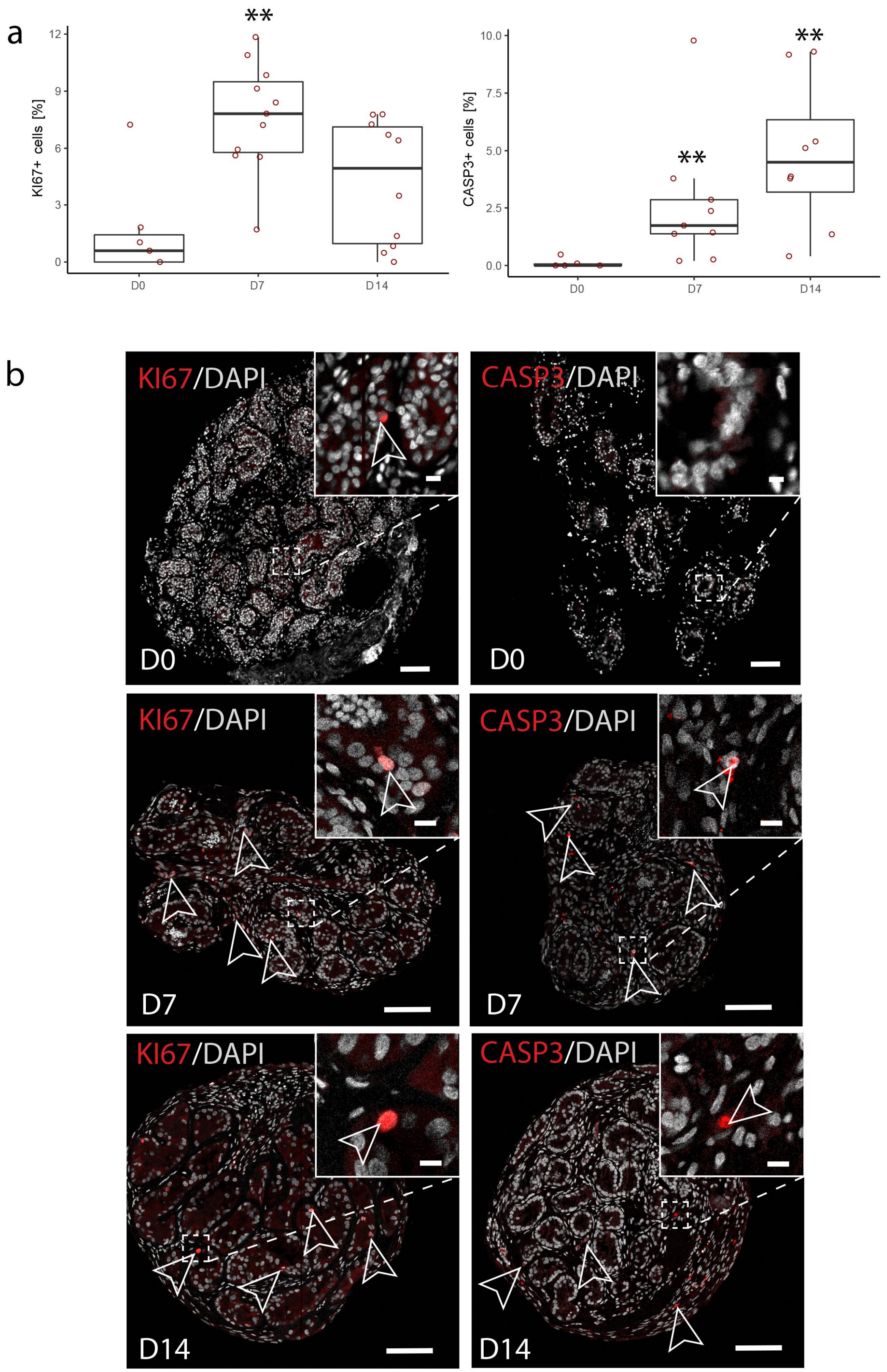

Figure S5: Increased expression of CASP3 and KI67 positive cells over 14 days in explant tissue culture of prepubertal testis samples (1.56 to 13.39 years of age). **a)** Percentage of KI67 stained prenatal samples at day 0 n=7, at day 7 n=11 and at day 14 n=10 as well as percentage of CASP3 stained prenatal samples at day 0 n=6, at day 7 n=9 and at day 14 n=8. ** indicate p <0.01 significance between day 0

and day 7, or day 0 and day 14. **b)** Representative images of KI67 (red staining) and CASP3 (red staining) expression in prepubertal testicular tissue samples at day 0 (D0), day 7 (D7) and day 14 (D14) is indicated in selected cells with arrows. Counterstain showing with DAPI (grey staining). Scale bars: 100µm, zoom scale bars: 10µm

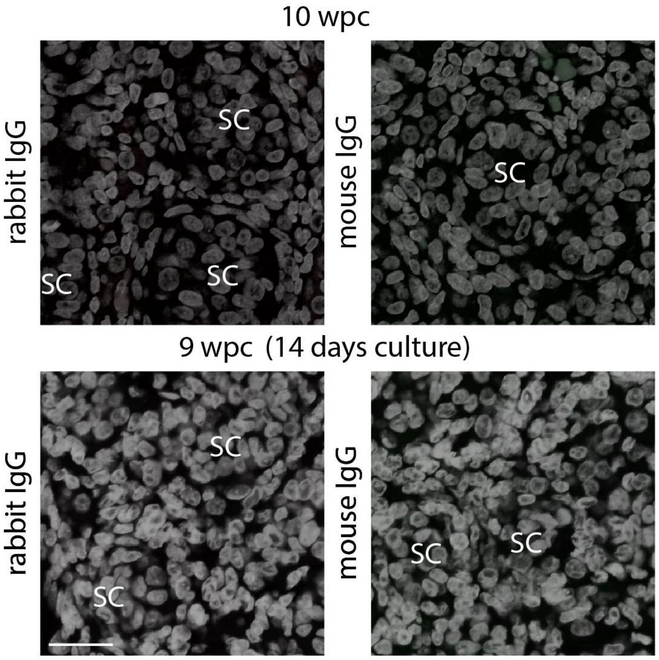

Figure S6: Negative IgG control for uncultured and cultured prenatal gonadal tissue. Counterstain with DAPI (grey staining). SC depicts the seminiferous cords, scale bar: 25µm

Table S1: Immunohistological evaluation of grouped prenatal male gonads

Abbreviation: wpc, weeks post conception. NT indicated samples without yet formed seminiferous boundaries and are shown as counting/total gonadal area; Light grey shading are 14 days cultured (C) gonads. Seminiferous cord area is expressed in mm².

wpc	OCT4^{+ve}/ seminiferous cord mm²	DDX4^{+ve}/ seminiferous cord mm²	MAGE-A4^{+ve}/ seminiferous cord mm²	seminiferous cord area / gonad in %
5-6NT	0.45 ±0.09	0.06 ±0.00	0.00	0.00
5-6T	1.09 ±0.31	0.47 ±0.44	0.01 ±0.02	28.4 ±7.31
6C	0.12	0.04	0.02	31.02
7-8	0.91 ±0.25	0.69 ±0.54	0.03 ±0.01	36.82 ±5.41
7C	0.76	0.21	0.21	9.15
9-10	1.12 ±0.83	1.38 ±1.27	0.03 ±0.03	22.06 ±8.71
9C	0.81	0.95	0.3	18.34
12-13	0.67 ±0.83	1.54 ±0.81	0.2 ±0.12	26.16 ±8.98
14-15	0.12 ±0.19	1.34 ±0.58	0.31 ±0.19	30.25 ±6.00
16-17	0.24 ±0.17	1.04 ±0.38	0.28 ±0.21	29.13 ±6.07

Table S2: Samples and immunohistological evaluation of individual prenatal gonadal tissue

Abbreviation: wpc, weeks post conception. Seminiferous cord area is expressed in mm^2. Vessel count is based on type IV collagen positive vessels.

Sample	wpc	OCT4^{+ve}/ seminiferous cord mm^2	DDX4^{+ve}/ seminiferous cord mm^2	MAGE-A4^{+ve}/ seminiferous cord mm^2	seminiferous cord area / gonad in %	Coll4^{+ve} blood vessel/gonad mm^2
E1	5	0.39	0.06	0.00	0	0.00
E2	5	0.74	0.41	0.03	23	0.12
E3	6	1.33	0.94	0.00	26	0.23
E4	6	0.51	0.07	0.00	0	0.00
E5	6	1.18	0.07	0.00	37	0.04
E6	7	0.98	0.18	0.02	42	0.23
E7	7	0.95	0.68	0.04	44	0.26
E8	8	1.30	1.62	0.02	37	0.29
E9	8	0.58	0.42	0.03	31	0.20
E10	8	0.73	0.99	0.05	30	0.27
E11	8	0.92	0.26	0.03	37	0.10
E12	9	2.27	3.22	0.06	33	0.24
E13	9	0.51	0.44	0.04	12	0.05
E14	9	0.51	0.68	0.01	24	0.19
E15	10	1.17	1.18	0.01	20	0.35
F1	12	0.00	2.44	0.33	31	0.29
F2	12	0.41	0.86	0.09	16	0.23
F3	13	1.60	1.32	0.19	32	0.17
F4	14	0.06	1.81	0.11	27	0.35
F5	15	0.44	1.69	0.43	24	0.13
F6	15	0.10	1.79	0.59	31	0.16
F7	15	0.00	0.73	0.20	40	0.18
F8	15	0.00	0.67	0.24	30	0.29
F9	16	0.12	0.67	0.27	24	0.19
F10	17	0.44	1.03	0.08	36	0.22
F11	17	0.16	1.42	0.49	28	0.29

Table S3: Immunohistological evaluation of individual postnatal control and cultured gonads

Abbreviation: D indicates days of culture; CED, cumulative cyclophosphamide equivalent dose; DIE, doxorubicin isotoxic dose equivalent; PID, primary immune deficiency; AML, acute myeloid leukaemia; ALL, acute lymphoblastic leukaemia, MDS, myelodysplastic syndrome. Germ cell quantification is expressed as positive cells per seminiferous tubule cross-section.

	Age	Diagnosis			DXX4+ve / round seminiferous tubules			% LAMA1+ve seminiferous tubules			% LAMA5+ve seminiferous tubules		
**Patient**	**Years**		**CED**	**DIE**	**D0**	**D7**	**D14**	**D0**	**D7**	**D14**	**D0**	**D7**	**D14**
P1	1.56	PID	0.00	0.00	0.49	0.08	0.27	64	0	0	0	0	0
P2	1.96	PID	0.00	0.00	0.46	0.63	0.00	100	35	0	0	0	N.A.
P3	2.35	Thalassemia	0.00	0.00	0.21	0.10	0.03	N.A.	83	60	0	82	27
P4	2.86	Neuroblastoma	0.00	0.00	0.55	0.26	0.00	100	100	20	0	N.A.	93
P5	3.01	PID	0.00	0.00	0.20	0.04	0.00	100	13	0	0	0	0
P6	3.77	AML	0.00	300.00	1.05	0.27	0.64	0	N.A.	N.A.	0	0	0
P7	5.34	Leukodystrophy	0.00	0.00	2.08	1.25	2.36	100	0	0	0	55	0
P8	6.72	ALL	0.00	109.00	0.93	0.50	0.00	100	N.A.	0	0	56	0
P9	7.24	MDS	0.00	0.00	1.91	1.12	1.00	100	38	21	0	0	88
P10	9.33	Thalassemia	0.00	0.00	0.73	N.A.	0.00	18	N.A.	0	0	0	0
P11	10.60	ALL	0.00	80.00	3.28	0.13	0.00	N.A.	0	0	0	0	0
P12	10.63	Aplastic anaemia	0.00	0.00	0.10	0.22	0.40	99	38	11	0	0	0
P13	11.74	AML	0.00	N.A.	1.89	0.22	0.00	89	60	20	0	0	0
P14	11.89	AML	0.00	300.00	2.10	2.11	0.25	N.A.	0	0	0	30	0
P15	11.93	ALL	0.00	80.00	1.54	0.00	0.08	67	N.A.	0	0	N.A.	54
P16	13.39	PID	0.00	0.00	7.21	3.38	0.00	82	75	100	0	0	0
Mean	7.15				1.55	0.69	0.31	78	37	16	0	16	17

Table S4: Primary and secondary antibodies.

Protein	Host	Antibody concentration	Antibody dilution	Catalogue number
**LAMA1**	mouse	0.7 mg/ml	1:20	ab210954
**LAMA2**	rabbit	N.A.	1:100	ab140482
**LAMA3**	mouse	0.5 mg/ml	1:80	AMAb91123
**LAMA4**	rabbit	N.A.	1:100	ab209675
**LAMA5**	mouse	1 mg/ml	1:200	ab77175
**Type I collagen**	rabbit	1 mg/ml	1:20	ab34710
**Type IV collagen**	rabbit	0.511 mg/ml	1:600	ab214417
**Type VI collagen**	rabbit	1 mg/ml	1:500	ab6588
**Fibronectin**	rabbit	0.058 mg/ml	1:200	ab32419
**POU5F1**	rabbit	1 mg/ml	1:200	ab19857
**DDX4**	mouse	1 mg/ml	1:200	ab13840
**DDX4**	rabbit	1 mg/ml	1:200	ab27591
**MAGE-A4**	mouse	N.A.	1:200	gift from Giulio Spagnoli
**Mouse IgG**	mouse	0.4 mg/ml	1:100	sc-2025
**Rabbit IgG**	rabbit	1.775 mg/ml	1:200	ab172730
